# Magnetically induced transparency of a quantum metamaterial composed of twin flux qubits

**DOI:** 10.1038/s41467-017-02608-8

**Published:** 2018-01-11

**Authors:** K. V. Shulga, E. Il’ichev, M. V. Fistul, I. S. Besedin, S. Butz, O. V. Astafiev, U. Hübner, A. V. Ustinov

**Affiliations:** 1 Physikalisches Institut, Karlsruhe Institute of Technology, D-76131 Karlsruhe, Germany; 2 Russian Quantum Center, National University of Science and Technology MISIS, Moscow, 119049 Russia; 3 Moscow Institute of Physics and Technology, Dolgoprudny, 141700 Moscow region Russia; 4 Leibniz Institute of Photonic Technology, PO Box 100239, D-07702 Jena, Germany; 5 Center for Theoretical Physics of Complex Systems, Institute for Basic Science, Daejeon, 34051 Republic of Korea; 6 Department of Physics, Royal Holloway, University of London, Egham, Surrey TW20 0EX UK

## Abstract

Quantum theory is expected to govern the electromagnetic properties of a quantum metamaterial, an artificially fabricated medium composed of many quantum objects acting as artificial atoms. Propagation of electromagnetic waves through such a medium is accompanied by excitations of intrinsic quantum transitions within individual meta-atoms and modes corresponding to the interactions between them. Here we demonstrate an experiment in which an array of double-loop type superconducting flux qubits is embedded into a microwave transmission line. We observe that in a broad frequency range the transmission coefficient through the metamaterial periodically depends on externally applied magnetic field. Field-controlled switching of the ground state of the meta-atoms induces a large suppression of the transmission. Moreover, the excitation of meta-atoms in the array leads to a large resonant enhancement of the transmission. We anticipate possible applications of the observed frequency-tunable transparency in superconducting quantum networks.

## Abstract (web summary)

Here, the authors demonstrate an array of superconducting qubits embedded into a microwave transmission line. They show that the transmission through the metamaterial periodically depends on externally applied magnetic field and suppression of the transmission is achieved through field-induced transitions.

## Introduction

Quantum metamaterials, media built from quantum objects acting as meta-atoms, promise to display novel light-matter interaction phenomena^1–9^. Nonclassical electrodynamics of such media is linked to many-body quantum mechanics of quantum simulators^10–12^, ensemble quantum memory^13,14^, generation of non-classical light states^15,16^, etc. The only reported to date experiments with many superconducting meta-atoms in the quantum regime employed arrays of superconducting qubits weakly coupled to a microwave resonator^17–19^. The observed variations of the transmission coefficient $\left| {S_{21}} \right|^2$ were, in that case, rather small and limited to a narrow frequency range.

In this work, we aim at achieving tunable electromagnetic properties of the medium in a wide frequency range, not limited by the qubit–resonator interaction. Here we demonstrate intriguing electrodynamic properties of a superconducting quantum metamaterial fabricated from an array of meta-atoms, each consisting of a pair of superconducting loops coupled via a tunnel junction (twin flux qubit). Such meta-atoms provide a strong coupling between qubits and propagating electromagnetic waves. The peculiarity of the twin qubit structure is the field induced 0 → *π* transition of the Josephson junction phases that leads to an abrupt suppression of the microwave transmission in a broad frequency range. In a narrow frequency range, we observe a great enhancement of the microwave transmission. Such resonant transparency is controlled by an external magnetic field. The detailed quantitative analysis is in a good accord with measurements.

## Results

### Physical device and control

**Fig. 1:**
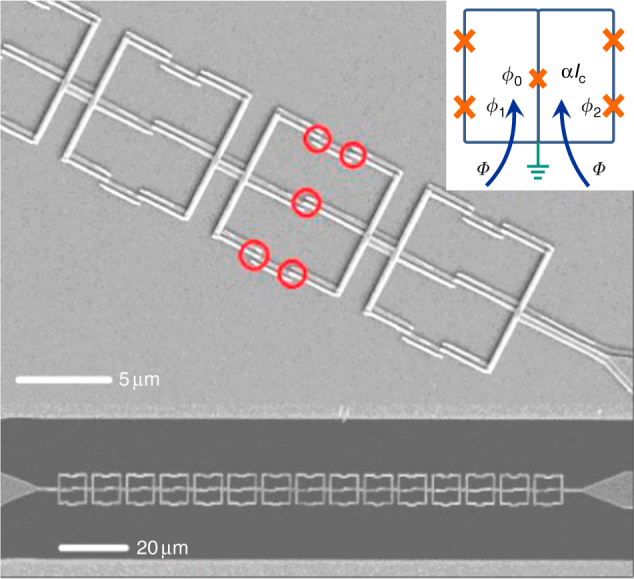
A quantum metamaterial made of twin flux qubits. Superconducting quantum metamaterial consisting of an array of 15 twin qubits embedded in a coplanar wave guide. An SEM image of twin flux qubits (above) and a whole structure (below) are shown. Each qubit consists of two superconducting loops sharing one common central Josephson junction (*α*-junction) and four identical Josephson junctions located on the outer parts of the loops. The *α*-junction allows the magnetic flux to tunnel between the loops. The inset is a schematic of a single meta-atom—the twin flux qubit; the phases on nodes are shown

The studied system consists of an array of 15 twin flux qubits, each of them containing five Josephson junctions (see Fig. 1). The central Josephson junction (called *α*-junction) of each qubit is shared by two superconducting loops and embedded directly into the central electrode of an Al coplanar waveguide (CPW). This setup provides an extremely large coupling between qubits and electromagnetic waves propagating through the system. We measured the microwave transmission through the CPW as a function of frequency and an external dc magnetic field applied perpendicular to the plane of the substrate. Experiments were performed at low power of the applied microwave field to remain in the linear driving regime. The radiation power is estimated to be on the order of −125 dBm at the chip input port.

### Twin flux qubit model

Precluding the following demonstration of the experimental data and its detailed theoretical analysis, we present the essence of our idea to implement a metamaterial based on twin flux qubit. The twin flux qubit circuit has four nodes. By grounding one of the nodes (setting its nodal phase to zero) and assigning nodal phase variables to the rest of them we can completely describe the state of the system. The Hamilton’s function, which is sum of the charge and Josephson energies, reads

1 $$\begin{array}{l}H(\phi ,{{q}}) = \frac{{(2e)^2}}{2}{{q}}\hat {{C}}^{ - 1}{{q}}^T + E_{\mathrm{J}}\left( {4 + \alpha - \alpha \,{\mathrm{cos}}\,\phi _0} \right.\\ \left. { - {\mathrm{cos}}\,\phi _1 - {\mathrm{cos}}\,\phi _2 - {\mathrm{cos}}\left( {\phi _2 - \phi _0 + \phi } \right) - {\mathrm{cos}}\left( {\phi _1 - \phi _0 - \phi } \right)} \right)\end{array}$$

where $\phi = \left( {\phi _0,\phi _1,\phi _2} \right)$ are the nodal phases and *q* = (*q*_0_, *q*_1_, *q*_2_) are the corresponding canonically conjugate charges (see inset Fig. 1). Here, *Φ* is the external flux applied to a single loop of the twin qubit. Since the Josephson energy is periodic in *Φ*, we analyze the phase distribution across the twin qubit structure at applied fluxes *Φ* from 0 to *Φ*_0_ per single loop of the twin-qubit, where *Φ*_0_ = *h*/2*e* is the magnetic flux quantum. When *Φ* is close to 0, the phase across the central junction *ϕ*_0_ is zero. Simple calculation demonstrates that at *Φ* close to *Φ*_0_/2 the minimum energy corresponds to *ϕ*_0_ = *π*. This result follows from phase quantization of the superconducting order parameter around the loop. Therefore, one can expect a sharp change of the transmission coefficient $\left| {S_{21}} \right|^2$ at the critical value of *Φ*_cr_ (between 0 and *Φ*_0_/2), and a jump back at the external flux ${{\Phi }} \simeq {{\Phi }}_0 - {{\Phi }}_{{{{\rm cr}}}}$. The value of *Φ*_cr_ depends on circuits parameters.

**Fig. 2:**
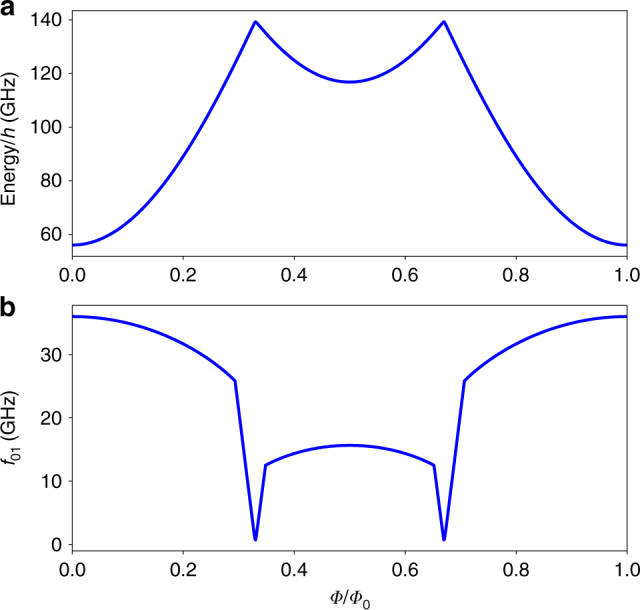
Magnetic field dependence of the transition frequencies of the twin qubit. The energy of the ground state (**a**) and the transition energy *hf*_01_ of the twin qubit calculated from the Hamiltonian of Eq. (1) (**b**). The parameters *α* = 0.72 and *C* = 5.2 fF and the Josephson energy is *E*_*J*_ = 50 GHz. These dependencies are *Φ*_0_ periodic and symmetric with respect to *Φ*/*Φ*_0_ = 0.5. The minimum point of the (**b**) plot corresponds to the transition of the central junction phase *φ*_0_ from zero to *π*

Replacing the nodal phase and charge variables by the respective operators in Hamilton’s function (see Eq. (1)) yields the Hamiltonian operator. By making use of numeric diagonalisation of the Hamiltonian the eigenvalues and the eigenstates are found (see Supplementary Note 1). The magnetic field dependence of the ground state energy of a twin flux qubit is presented in Fig. 2a. For the chosen experimental parameters of the twin flux qubit, in the vicinity of zero-applied flux, the ground-excited state transition energy *hf*_01_ is quite large (see Fig. 2b). However, if the external flux is close to the value of *Φ*_0_/2 per each loop, the energy *hf*_01_ is about 10 GHz and has an extremum (“sweet spot”). At this point, the twin-qubit transition energy reaches a local maximum rather than minimum characteristic for the standard single-loop three-junction flux qubit^20^. Moreover, the flux dependence of *f*_01_ is much weaker than that of the usual flux qubit. This makes the twin-qubit more immune to the well known flux qubit problem—strong sensitivity of the transition energy to the external magnetic field fluctuations leading to unavoidable dephasing.

### Measurements of the Transmission Coefficient

**Fig. 3:**
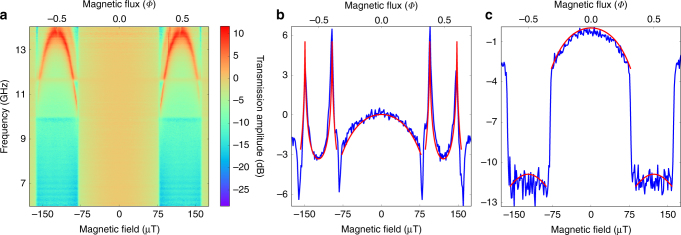
Transmission of microwaves through the quantum metamaterial in different regimes. **a** The measured dependence of the amplitude of transmission coefficient *t* (normalized to the value at zero field) on applied dc magnetic field (proportional to the bias current in the coil, lower axis) and frequency *f*. The upper horizontal axis translates the field in magnetic flux *Φ* per qubit single loop. The transmission *t* displays the sharp changes under variation of the magnetic flux *Φ*. One can see two different ranges of microwave propagation, nearly flat transmission around zero field and sharp resonant enhancement of the transmission near 11–14 GHz at magnetic flux *Φ* ~ ±*Φ*_0_/2. **b** A cross-cut of **a** at the fixed frequency of 13 GHz. The sharp peaks correspond to coherent tunneling between quantum states in the twin qubits (see text). **c** A cross-cut of **a** at the fixed frequency of 10 GHz. The sharp jumps correspond to a transition between zero and *π* phase on the central junction of the twin qubit (see text). Red curve is a fit to the theoretically predicted dependence Eq. (12)

The measured dependence of the transmission coefficient $\left| {S_{21}} \right|$ on both the microwave frequency *f* and the externally applied magnetic flux is shown in Fig. 3a. First, as it is expected, one notices that $\left| {S_{21}} \right|$ is symmetric around zero field and periodic with respect to the external magnetic flux *Φ* (outside the range of Fig. 3) with period of *Φ*_0_ per single qubit loop. Second, a spectacular result of the measurement is the presence of two regions in the magnetic flux with drastically different transmission coefficients $\left| {S_{21}} \right|$, see the orange and blue areas in Fig. 3a. The transition between these two regimes is abrupt and occurs at magnetic flux values *Φ*_cr_ ≃ ±0.3*Φ*_0_, see Fig. 3c. In the range 0.3*Φ*_0_ ≤ *Φ* ≤ 0.7*Φ*_0_, the transmission amplitude $\left| {S_{21}} \right|$ changes weakly (from −10 to −5 dB) in frequency band 6–14 GHz outside the resonance (the red feature). The intriguing field-dependent resonant features are characterized by a large enhancement of the transmission amplitude (appearing in Fig. 3a in red) over a relatively narrow frequency range between 11 and 14 GHz. The two symmetric resonant features reach their maximum frequency near *Φ* ~ ±*Φ*_0_/2. The induced resonant transmission frequency is smoothly decreasing on either side from the maximum and disappears at around 11 GHz.

In order to understand qualitatively the observed modulation of the microwave transparency, let us consider the dependence of the qubit state on the applied magnetic field. At low temperatures, the metamaterial is in its ground state. Therefore, the phases across qubit junctions can be reconstructed as functions of magnetic fluxes applied through qubit loops. Since the external magnetic flux is applied symmetrically to the twin qubits structure, induced current across central junction is zero, and its phase, consequently, is either zero or *π*. Along with increasing the applied magnetic flux, a circulating current through external junctions is induced, providing a monotonic increase of the Josephson phases of the outer Josephson junctions and, in turn, the effective qubit inductance. This causes a smooth decrease of the transparency, see Fig. 3c.

As soon as the external magnetic flux reaches the value of *Φ*_cr_, it is energetically favorable to increase the energy of the central junction and to lower the energies of the outer junctions. Quantitatively this change is dictated by the generalized Bohr–Sommerfeld quantization rule ensuring a single-valued wave-function of the condensate across qubits loops. As a result at $\left| {{\Phi }} \right| = {{\Phi }}_{{\mathrm{cr}}}$ the phase shift of the central junction *ϕ*_0_ from zero to *π* is accompanied by the phase shift of the outer junctions by *π*/2, see Supplementary Fig. 4, and the transmission amplitude sharply drops, see Fig. 3c. We call the ground state of the twin qubit in this magnetic field range the *π*-state as opposed to the zero-state at $\left| {{\Phi }} \right| < {{\Phi }}_{{\mathrm{cr}}}$. In the *π*-state, the currents in the outer branches of the twin flux qubit also change according to the new values of the phases. In particular, at the “sweet” spot *Φ* = *Φ*_0_/2 phases of all five Josephson junctions of the twin qubit are either zero or *π* which implies zero currents flowing through all junctions, causing the absence of current flowing in all branches of the twin flux qubit.

This nonresonant effect is observed for the entire frequency range used in our experiments and caused by the field-dependent phase distribution in the twin-qubit metamaterial in its ground state. The experimentally obtained value of *Φ*_cr_ is consistent with the calculated dependence on *α*, see Supplementary Note 2. Important to note that widely discussed Electromagnetically Induced Transparency (EIT) has a different nature than the phenomenon described here. “Conventional” EIT is caused by the quantum interference between different scattering channels. It immediately demands the presence of many (at least three) energy levels for joint photon–qubit systems^21–26^. At contrary in the case considered here the classical electromagnetic field interacts with a regular array of qubits, and EIT occurs due to changing of the ground state of a twin flux qubits arrangement.

In the *π*-state region of external magnetic flux, the transition energy between the ground and first excited states of the twin qubit *hf*_01_ can be determined experimentally. The sharp peaks in Fig. 3b and inverted red parabolas between 11 and 14 GHz in Fig. 3a correspond to resonant enhancement of transmission amplitude resulting from the $\left| 0 \right\rangle \to \left| 1 \right\rangle$ transition. These experimental data in Fig. 3b are fitted by the transition frequency *f*_01_ obtained from diagonalisation of the Hamiltonian (1) and excellent agreement is observed, also see Supplementary Fig. 2.

### Analysis of the transmission coefficient

In spite of the quantum nature of the twin qubit and the strong coupling between qubits and applied microwaves, here we first describe the electromagnetic field as a classical wave. Below we quantitatively analyze the wave propagation through the transmission line which is strongly inductively coupled to a periodic array of quantum oscillators. The transmission line is characterized by coordinate and time dependent charge distribution *Q*(*x*, *t*). Here, *x* is the coordinate along the transmission line. In the presence of a periodic array of twin qubits, which can be considered as superconducting SQUID-like oscillators, the electromagnetic wave propagation is determined by the equation as

2 $$c_0^2\frac{{\partial ^2Q}}{{\partial x^2}} - \frac{{\partial ^2Q}}{{\partial t^2}} - \gamma _{{\mathrm{tl}}}\frac{{\partial Q}}{{\partial t}} + \mathop {\sum}\limits_n \beta \left\{ {Q\left( {x_n,t} \right)} \right\}\delta \left( {x - x_n} \right) = 0$$

where the parameter *β*{*Q*(*x*_*n*_, *t*)} is the effective scattering strength of a single twin qubit located at *x* = *x*_*n*_. The parameter *γ*_tl_ describes the intrinsic dissipation of the transmission line (*γ*_tl_ ≪ 1), and *c*_0_ is the velocity of electromagnetic waves in the transmission line.

In the particular case of a twin flux qubit (see Fig. 1) the parameter *β* is obtained from the quantum-mechanical average of the time-dependent Josephson phase of the *α*-junction *φ*_0_(*t*), i.e.,

3 $$\beta = \frac{\hbar }{{2eL}}\left\langle {\dot \phi _0(t)} \right\rangle$$

where *L* is the transmission line inductance per unit length. The quantum dynamics of the Josephson phase *ϕ*_0_ is determined by the stationary Hamiltonian (1) and the time-dependent perturbation Hamiltonian, $H_{{\mathrm{per}}} = \left( {E_{\mathrm{J}}{\mathrm{/}}I_{\mathrm{c}}} \right)\dot Q(x,t)\phi _0$.

Let us consider an electromagnetic wave of a given frequency *ω*, *Q*(*x*,*t*) = *Q*(*x*)*e*^*iωt*^. In this case, Eq. (2) can be re-written as

4 $$c_0^2\frac{{\mathrm{d}^2Q(x)}}{{\mathrm{d}x^2}} + \left( {\omega ^2 - \gamma _{{\mathrm{tl}}}i\omega } \right)Q(x) + \mathop {\sum}\limits_n \beta \left\{ {Q\left( {x_n} \right)} \right\}\delta \left( {x - x_n} \right) = 0$$

Following the method elaborated for the solution of the Schrödinger equation with the Kronig–Penney potential^27^, we can present the charge distribution *Q*(*x*) in the following form:

5 $$Q(x) = - \frac{i}{{2k}}\mathop {\sum}\limits_n \beta \left\{ {Q_n} \right\}{\mathrm{exp}}\left( {ik\left| {x - x_n} \right|} \right)$$

where the wave vector $k = \sqrt {\omega ^2 + i\gamma _{{\mathrm{tl}}}\omega } {\mathrm{/}}c_0$ and *Q*_*n*_ = *Q*(*x*_*n*_) is the amplitude of propagating charge distribution at point *x*_*n*_. By making use of the properties of the *δ*-function, we obtain a set of discrete equations for *Q*_*n*_:

6 $$Q_{n + 1} + Q_{n - 1} - 2\left[ {Q_n\,{\mathrm{cos}}\,ka - \frac{{\beta \left\{ {Q_n} \right\}}}{{2kc_0^2}}{\mathrm{sin}}(ka)} \right] = 0$$

Here, *a* is the effective distance between adjacent point-type scatterers of the array. Taking into account that, in the microwave range, $ka \ll 1$, we substitute the discrete values of *Q*_*n*_ with a smooth function $\tilde Q(x)$ satisfying the equation

7 $$\frac{{{\rm d}^2\tilde Q(x)}}{{\mathrm{d}x^2}} + k^2\tilde Q(x) - \frac{{\beta \left\{ {\tilde Q(x)} \right\}}}{{ac_0^2}} = 0$$

For low amplitude of the waves, we obtain an explicit expression for the effective scattering strength *β*:

8 $$\beta = - \frac{{\hbar \omega ^2}}{{2eLI_{\mathrm{c}}}}K(\omega )Q,K(\omega ) = \frac{{\omega _{\mathrm{p}}^2}}{{{{\Omega }}^2 - \omega ^2 + i\gamma _{\mathrm{J}}}}$$

where *Ω* = Δ*E*/*ħ* is the characteristic frequency of the oscillator that can be used to approximate the lowest-lying energy levels of the twin flux qubit. For small external flux values $\left| {{\Phi }} \right| < {{\Phi }}_{{\mathrm{cr}}}$, where *Φ*_cr_ = *Φ*_0_/*π* arcsin *α*, the 0-state is the unique stable state of the twin qubit. For the qubits with *α* value used in this paper, *Φ*_cr_ ≈ 0.3*Φ*_0_. The characteristic frequency *Ω* = *Ω*_+_ in the range $\left| {{\Phi }} \right| < {{\Phi }}_{{\mathrm{cr}}}$ is relatively large (see Fig. 2b).

9 $${{\Omega }}_ + ^2 = \omega _{{p}}^2\frac{{{\mathrm{cos}}\left[ {\pi {{\Phi /\Phi }}_0} \right] + \alpha }}{{1 + \alpha }}$$

Notice here, that the frequency *Ω*_+_ smoothly decreases with the applied magnetic flux *Φ* and results in a parabolic non-resonant decrease of $\left| {S_{21}} \right|$ as observed in experiments, see the central part of the $\left| {S_{21}} \right|$ curve in Fig. 3b.

The characteristic frequency of small-amplitude quantum oscillations in the *π*-state for ${{\Phi }}_{{\mathrm{cr}}} < \left| \Phi \right| < {{\Phi }}_0 - {{\Phi }}_{{{{\rm cr}}}}$ can be written explicitly as (see the central part of Fig. 2b).

10 $${{\Omega }}_ - ^2 = \omega _{{p}}^2{\kern 1pt} \frac{{{\mathrm{sin}}\left[ {\pi {{\Phi /\Phi }}_0} \right] - \alpha }}{{1 + \alpha }}$$

Since the characteristic frequency ${{\Omega }}_ - \ll {{\Omega }}_ +$ one expects strong changes of the magnitude of the transmission coefficient $\left| {S_{21}} \right|$ at magnetic flux values *Φ* = *Φ*_cr_ and *Φ* = *Φ*_0_ − *Φ*_cr_.

Substituting (8) in (7) we obtain an equation for the propagation of electromagnetic waves in this quantum metamaterial:

11 $$\frac{{{\rm d}^2\tilde Q(x)}}{{\mathrm{d}x^2}} + k^2[1 + AK(\omega )]\tilde Q(x) = 0$$

where the parameter *A* ≈ *ħ*/(2*eLaI*_c_) depends on the properties of both the transmission line and the Josephson junctions. In our case $A = 400 \gg 1$ and in this limit we can obtain the transmission coefficient $S_{21} = \left| {Q(l){\mathrm{/}}Q(0)} \right|$ (*l* is the length of a transmission line) in the following form:

12 $$\left| {S_{21}} \right| = \left| {{\mathrm{cos}}\left( {kl\sqrt {AK(\omega )} } \right) + i\frac{{\sqrt {AK(\omega )} }}{2}{\mathrm{sin}}\left( {kl\sqrt {AK(\omega )} } \right)} \right|^{ - 1}$$

## Discussion

Equation (12) shows a strong dependence of the transmission coefficient $\left| {S_{21}} \right|$ on the characteristic frequency *Ω*. In particular, there is a non-resonant suppression of $\left| {S_{21}} \right|$ due to a great enhancement of *K*(*ω*) in the 0-state (see Eq. (8)). However, as *ω* gets close to the *Ω*_−_, the resonant condition is satisfied, i.e., $kl\left| {\sqrt {AK(\omega )} } \right| = \pi$, and the qubit array becomes transparent. The typical magnetic field dependence of *S*_21_ for two different frequencies is shown by red lines in Fig. 3b, c. We stress here that in order to observe the both physical effects, the two essential conditions has to be satisfied, namely, a large coupling $\left( {A \gg 1} \right)$ between qubits and the transmission line, and a large number of qubits in the metamaterial $\left( {l{\mathrm{/}}a \gg 1} \right)$.

The resonant propagation of electromagnetic field has to be rather sensitive to the disorder in qubits parameters, and therefore, the observation of a single large resonance in the dependence of *S*_21_ on magnetic field, strongly indicates the presence of collective behavior in systems of many qubits.

We have presented the results of the study of the microwave transmission through the quantum metamaterial composed of twin flux qubits. The measured transmission spectra demonstrate that the transparency of the metamaterial is controlled by the external magnetic field in a broad frequency range. Observed large resonant enhancement of the transmission coefficient of the metamaterial in a relatively narrow frequency range corresponds to the qubit $\left| 0 \right\rangle$ → $\left| 1 \right\rangle$ transition. Additionally, an abrupt drop of the transmission induced by the external magnetic field and observed in a wide frequency range is caused by the 0 → *π* transition of the *α*-junction, corresponding to a change of the system ground state.

## Methods

### Sample fabrication

The Josephson junctions were fabricated by the shadow-evaporation technique using thin-film Al with a AlO_*x*_ tunnel barrier. The four junctions placed on the outer sides of the superconducting loops have the size of 200 × 355 nm^2^ each, and the central Josephson junction is by a factor *α* = 0.72 smaller. The resulting outer Josephson junctions are estimated to have a Josephon energy of the standard junctions *E*_J_ = 39 × *h* GHz. The ratio of the Josephson and charging energy of *α*-junction is *E*_J_/*E*_C_ ≈ 3.8.

### Experimental setup

All measurements were carried out at a temperature of about 20 mK in a dilution refrigerator. The frequency dependent microwave transmission coefficient $\left| {S_{21}} \right|$ was measured with a vector network analyzer.

A superconducting coil around the sample holder was employed to apply a homogeneous dc magnetic field perpendicular to the sample plane. We calibrated the applied dc magnetic field in units of the magnetic flux quantum *Φ*_0_ penetrating the individual qubit loops by making use of similar experiments performed with a single-loop reference SQUID. Ground planes and contact pads on the chip were made out of gold in order to minimize flux focusing and reduce flux noise produced by creeping Abrikosov vortices.

### Data availability

The data that support the findings of this study are available from the corresponding author on request.

## Electronic Supplementary Material

Supplementary Information
