# Supplementary material for: Magnetically induced transparency of a quantum metamaterial composed of twin flux qubits
Source: Nat Commun. 2018 Jan 11;9:150. doi: 10.1038/s41467-017-02608-8 (PMC5764976; doi:10.1038/s41467-017-02608-8)
Supplement: Supplementary file 1 — Supplementary Information [file 41467_2017_2608_MOESM1_ESM.pdf]

SUPPLEMENTARY NOTE 1: NUMERICAL DIAGONALIZATION OF THE TWIN QUBIT HAMILTONIAN

In order to validate the theoretical ansatz of the quantum energy level calculation for the twin qubit described in the article, below we numerically diagonalize the qubit Hamiltonian to obtain the frequencies of the transitions between the energy levels of the qubit.

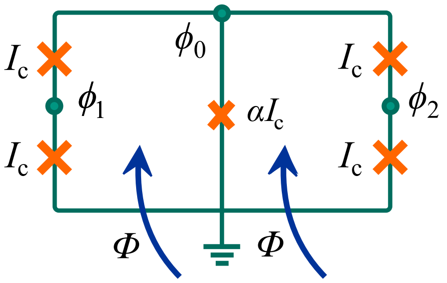

FIG. 1: *

Supplementary Figure 1: **Lumped element qubit schematic used for quantization.** The external fluxes are replaced by equivalent dc current sources.

We assume that the Josephson energies and capacitances of the junctions scale proportionally with their area. The four outer Josephson junctions have the same capacitances C and the Josephson energies E_J, while the central junction's capacitance and Josephson energy are αC and αE_J, respectively. For the qubit circuit, we define the nodal phases $\boldsymbol{\phi} = (\phi_0, \phi_1, \phi_2)$ at the nodes 0, 1 and 2 as shown in Fig. 1. Their time derivatives $\dot{\vec{\phi}}$ are proportional to the nodal voltages. For notational convenience, we introduce the dimensionless external flux through each of the qubit loops as $\phi = 2\pi\frac{\Phi}{\Phi_0}$. Following Devoret's circuit quantization procedure [1] we write the circuit's Lagrange function as

$$L(\boldsymbol{\phi}, \dot{\boldsymbol{\phi}}) = \frac{(2e)^2}{2\hbar^2}\dot{\boldsymbol{\phi}}\hat{\boldsymbol{C}}\dot{\boldsymbol{\phi}}^T - E_J(4 + \alpha - \alpha\cos\phi_0 - \cos\phi_1$$
$$\cos\phi_2 - \cos(\phi_2 - \phi_0 + \phi) - \cos(\phi_1 - \phi_0 - \phi))\ , \quad (1)$$

where the capacitace matrix $\hat{\boldsymbol{C}}$ is given by

$$\hat{C} = C\begin{pmatrix} 2+\alpha & -1 & -1 \\ -1 & 2 & 0 \\ -1 & 0 & 2 \end{pmatrix} . \quad (2)$$

Hamilton's function can be obtained by performing a Legendre transform of the Lagrange function with respect to the nodal charge $\boldsymbol{q} = (q_0, q_1, q_2)$ and voltage $\dot{\boldsymbol{\phi}}$ pairs:

$$H(\boldsymbol{\phi}, \boldsymbol{q}) = \frac{(2e)^2}{2}\boldsymbol{q}\hat{\boldsymbol{C}}^{-1}\boldsymbol{q}^T + E_J(4 + \alpha - \alpha\cos\phi_0$$
$$-\cos\phi_1 - \cos\phi_2 - \cos(\phi_2 - \phi_0 + \phi) - \cos(\phi_1 - \phi_0 - \phi)) \quad (3)$$

Replacing the phase and node variables by the respective operators in Hamilton's function yields the quantum Hamiltonian. Numerical simulation of the energy levels is performed by discretization of the degrees of freedom and consequent diagonalization of the discretized Hamiltonian matrix. We obtain a few ($m = 3$) lowest-lying energy levels with the Arnoldi sparse diagonalization algorithm [2], which requires not the full Hamiltonian matrix to be stored in the computer memory but rather the action of the operator on a arbitrary vector to be efficiently implemented. This allows us to take advantage of a specific property of the Hamiltonian matrix: it can be divided into a kinetic part, which is diagonal in charge representation, and a potential part, which is diagonal in phase representation. We compute actions of these parts separately in their eigenbases, and transition between charge and phase representation is implemented with the fast Fourier transform. The complexity of the whole diagonalization procedure is determined by the cost of Fourier transforms. The asymptotic expression for the complexity is $O(dmn^{2d}\log n)$, where $d = 3$ is the number of degrees of freedom and n is the number of steps along each of the wave function variables, which is faster than the asymptotic complexity $O(n^{3d})$ of general-purpose eigensolvers like QR-decomposition.

The searched state space of the qubit was restricted to $[0, 2\pi]$ intervals along each of the three nodal phase variable axes discretized on an orthogonal grid with $n = 32$ steps along each axis. Two of the Hamiltonian parameters, C and α have been estimated with their respective design values 5.2 fF and 0.72, while the value of the Josephson energy $E_J = 39$ GHz has been extracted from the microwave transmission spectrum by fitting the transmission resonance to the transition energy between the ground and first excited states of the qubit. Results of the fitting are shown in Fig. 2 (a).

The sweet spot of twin qubit at $\Phi_0/2$ corresponds to an energy maximum, rather than a minimum as in a conventional flux qubit. Moreover, the twin qubit is less sensitive to flux noise than the conventional flux qubit due to the flatter dispersion curve. However, the anharmonicity of the twin qubit is significantly less than that of a flux qubit as can be seen by comparing the $|0\rangle \to |1\rangle$ and $|1\rangle \to |2\rangle$ transition energies in Fig. 2(b).

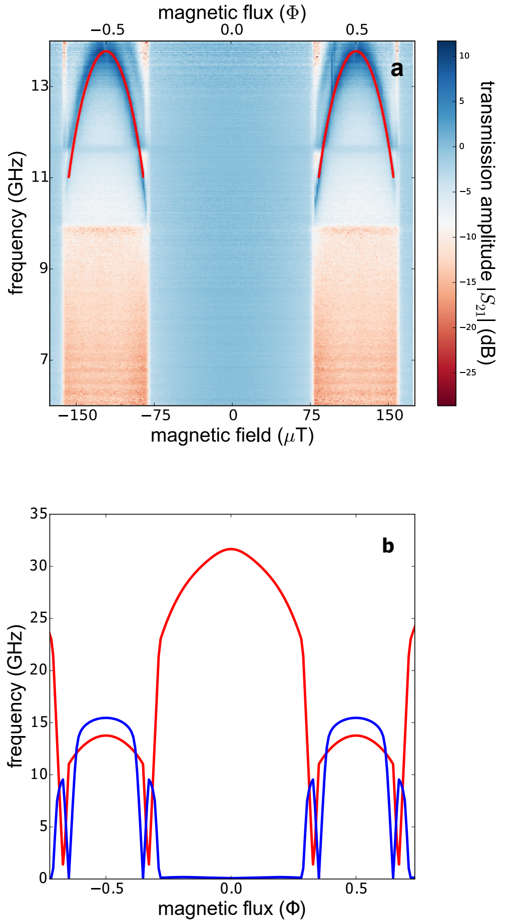

FIG. 2: *

Supplementary Figure 2: **Comparison of experimental spectrum with numerical diagonalization.** (a) Transmission spectrum (Fig. 3) fitted with the transition energy between the ground and first excited state of the twin qubit obtained by diagonalization of the qubit Hamiltonian (3). (b) Frequency of the transition between $|0\rangle \to |1\rangle$(red) and $|1\rangle \to |2\rangle$ (blue) quantum levels of twin qubit. Spectrum display two different regions occurring with the variation of the magnetic flux Φ. The sharp jumps correspond to the transition between classical metastable states of mirror flux qubits. The transition $E_{12} = 0$ at magnetic flux values $|\Phi| < \Phi_{\rm cr}$ due to degeneracy of the first and the second excited energy levels, which correspond to the clockwise and anticlockwise direction of the persistent current inside the twin qubit loop.

SUPPLEMENTARY NOTE 2: ANALYSIS OF THE JOSEPHSON JUNCTION PHASES DISTRIBUTION AT THE DIFFERENT TWIN FLUX QUBIT STATES

To explain the changes of the phases and the currents flowing through the Josephson junctions in the twin flux qubit with the external magnetic field changes, we write the total potential energy of this qubit. We denote phases on the junctions as shown in Fig. 1, taking into account the two magnetic flux quantization conditions in the qubit loops.

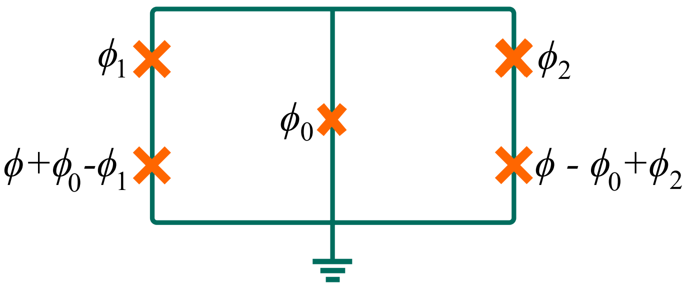

FIG. 3: *

Supplementary Figure 3: Phase distribution on the Josephson junctions of the twin flux qubit.

The full potential energy of the twin flux qubit can be expressed as the sum of the potential energy of each junction:

$$U = E_J(4 + \alpha - \alpha\cos(\phi_0) - \cos(\phi_1) - \cos(\phi_2) - \\ - \cos(\phi_2 - \phi_0 + \phi) - \cos(\phi_1 - \phi_0 - \phi)), \quad (4)$$

where $\phi = 2\pi\frac{\Phi}{\Phi_0}$ is the dimensionless external flux.

The stable states of the twin flux qubit correspond to extrema of this potential energy $\phi_0, \phi_1, \phi_2 = \operatorname{argmin} U$:

$$0 = \begin{cases} \frac{\partial U}{E_J\partial\phi_0} = \alpha\sin(\phi_0) - \sin(\phi - \phi_0 + \phi_2) + \\ + \sin(\phi + \phi_0 - \phi_1), \\ \frac{\partial U}{E_J\partial\phi_1} = \sin(\phi_1) - \sin(\phi + \phi_0 - \phi_1), \\ \frac{\partial U}{E_J\partial\phi_2} = \sin(\phi_2) - \sin(\phi - \phi_0 - \phi_2). \end{cases} \quad (5)$$

Depending on the parameter α, different sets of solutions corresponding to minima of the potential energy exist:

- A: $\phi_0 = 0$, $\phi_1 = -\frac{\phi}{2}$, $\phi_2 = \frac{\phi}{2}$,
- B: $\phi_0 = \pi$, $\phi_1 = \frac{\phi - \pi}{2}$, $\phi_2 = \frac{\pi - \phi}{2}$.
- C_1:
  $\phi_0 = 2\arcsin\left(\frac{1}{\alpha}\sin\frac{\phi}{2}\right)$,
  $\phi_1 = \arcsin\left(\frac{1}{\alpha}\sin\frac{\phi}{2}\right) - \frac{\phi}{2}$
  $\phi_2 = \arcsin\left(\frac{1}{\alpha}\sin\frac{\phi}{2}\right) + \frac{\phi}{2}$,
- C_2:
  $\phi_0 = -2\arcsin\left(\frac{1}{\alpha}\sin\frac{\phi}{2}\right)$,
  $\phi_1 = \pi - \arcsin\left(\frac{1}{\alpha}\sin\frac{\phi}{2}\right) - \frac{\phi}{2}$
  $\phi_2 = \pi - \arcsin\left(\frac{1}{\alpha}\sin\frac{\phi}{2}\right) + \frac{\phi}{2}$.

The dependance of the junction phases on applied magnetic flux is shown in Fig. 4.

At zero flux $\Phi = 0$, the phases on all five Josephson junctions of the qubit are zero, and the energy of the entire structure is minimal. As the magnetic flux increases,

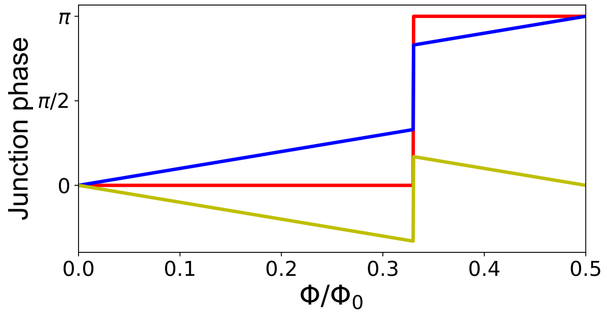

FIG. 4: *

Supplementary Figure 4: **Calculated dependence of the twin flux qubit junction phases on applied magnetic flux.** The sharp jumps at $\Phi = \Phi_{\rm cr}$ correspond to a 0 to π phase transition on the central junction of the twin qubit and to a change of the ground state of the system. Red, blue and yellow curves shows the phases on central and outer junctions on the magnetic flux respectively.

the phases and the energies of the outer junctions grow. The current through the central junction remains zero, because of mutual compensation of currents flowing in opposite directions in the qubit loops. At $\Phi_{\rm cr}$ it becomes favorably for the central junction to switch its phase to π, thereby increasing its energy and allowing junctions on the outer branches to reduce their energies. In order to satisfy the quantization condition for the flux in the loops at external junctions, the phase changes by $\pi/2$, which causes a current jump in the large qubit loop.

The solution A corresponds to the 0 - state of the twin flux qubit. Depending on α, the π - state is can be characterized by either a single-well (B) or symmetric double-well potential (C_1 and C_2). Due to quantum fluctuations of the phase, the transition from single to double well potential is gradual and doesn't lead to abrupt changes in the qubit properties. We emphasize that the transition between 0 - state (A) and double-well π - state (C_1 and C_2) is accompanied by the emergence of ground state degeneracy.

The value of $\Phi_{\rm cr}$ can be calculated by equating the energies of the solutions E_A and E_B:

$$E_A = 4E_J(1 - \cos\left(\frac{\phi}{2}\right)),$$

$$E_B = 4E_J(1 + \frac{1}{2}\alpha - \sin\left(\frac{\phi}{2}\right)),$$

from which $\Phi_{\rm cr}$ is given by:

$$\phi = \frac{2\pi\Phi_{\rm cr}}{\Phi_0} = \arcsin\left(1 - \frac{\alpha^2}{4}\right). \quad (6)$$

For the parameter $\alpha = 0.72$ used in the experiment, the value of the critical magnetic flux $\Phi_{\rm cr} \approx \Phi_0/3$, following from Eq. (6), agrees well with the experimental data (Fig. 3a). Similarly calculation can be done for the states C_1 and C_2. The stability diagram for all possible states of the twin flux qubit for different parameters α and Φ is shown in Fig. 5.

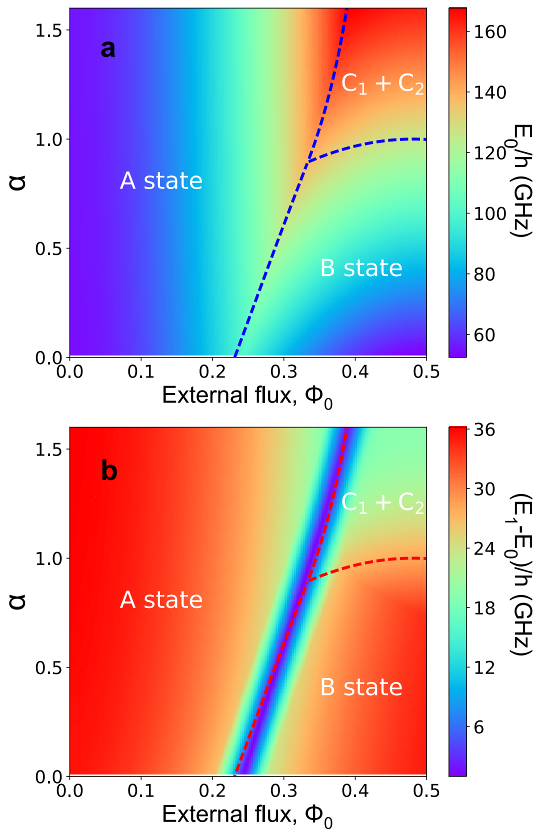

FIG. 5: *

Supplementary Figure 5: **Stability diagram of the twin flux qubit.** (a) ground state energy; (b) transition energy between the ground and the first excited state as a function of the external magnetic flux and α. The dashed lines show transition between different ground states.

Thus, the twin flux qubit transition from 0 to π state occurs in a wide frequency range, which is an important feature of this type of qubits, and is motivated by energy reasons.

SUPPLEMENTARY REFERENCES

[1] M. H. Devoret, in Quantum Fluctuations (Les Houches Session LXIII), edited by S. Reynaud, E. Giacobino, and J. Zinn-Justin (Elsevier, 1997), pp. 351386.

[2] W. E. Arnoldi, Q Appl. Math. Vol. 9, No. 1 (April, 1951), pp. 17-29
